# High-Performance Liquid Chromatography-Tandem Mass Spectrometry for Buprenorphine Evaluation in Plasma—Application to Pharmacokinetic Studies in Rabbits

**DOI:** 10.3390/molecules26020437

**Published:** 2021-01-15

**Authors:** Marta Tikhomirov, Błażej Poźniak, Tomasz Śniegocki

**Affiliations:** 1 Department of Pharmacology and Toxicology, Faculty of Veterinary Medicine, Wroclaw University of Environmental and Life Sciences, 50-375 Wrocław, Poland; zaklad.toksykologii@upwr.edu.pl; 2 Department of Pharmacology and Toxicology, National Veterinary Research Institute, 24-100 Pulawy, Poland; sekretariat@piwet.pulawy.pl

**Keywords:** buprenorphine, mass spectrometry, pharmacokinetics, rabbit

## Abstract

The precise and reliable determination of buprenorphine concentration is fundamental in certain medical or research applications, particularly in pharmacokinetic studies of this opioid. The main challenge is, however, the development of an analytical method that is sensitive enough, as the detected in vivo concentrations often fall in very low ranges. Thus, in this study we aimed at developing a sensitive, repeatable, cost-efficient, and easy HPLC analytical protocol for buprenorphine in rabbit plasma. In order to obtain this, the HPLC-MS^2^ system was used to elaborate and validate the method for samples purified with liquid-liquid extraction. Fragment ions 468.6→396.2 and 468.6→414.2 were monitored, and the method resulted in a high repeatability and reproducibility and a limit of quantification of 0.25 µg/L with a recovery of 98.7–109.0%. The method was linear in a range of 0.25–2000 µg/L. The suitability of the analytical procedure was tested in rabbits in a pilot pharmacokinetic study, and it was revealed that the method was suitable for comprehensively describing the pharmacokinetic profile after buprenorphine intravenous administration at a dose of 300 µg/kg. Thus, the method suitability for pharmacokinetic application was confirmed by both the good validation results of the method and successful in vivo tests in rabbits.

## 1. Introduction

Buprenorphine (BUP) is a semi-synthetic opioid exerting analgesic, sedative, and euphoric effects [1]. As a partial μ-receptor agonist and κ-receptor antagonist, it can be used to treat various types of pain (acute and chronic) in psychiatric disorders and to treat patients addicted to opioids [2]. Its use is widely established in many different formulations, including tablets and intravenous infusions, not only in human medicine but also in veterinary practice and preclinical studies on laboratory animals. In all these applications, the assessment of BUP concentration in biological samples may be of utmost importance, especially in cases of overdoses, poisonings, or when unauthorized use is suspected. The precise and reliable determination of drug concentration is fundamental in certain medical or research applications, particularly in pharmacokinetic (PK) studies. However, the main challenge in PK studies involving opioids lies in the need for a very sensitive analytical method able to evaluate low but clinically relevant drug concentrations in the nanogram range. The maximal concentrations of BUP reported in human PK studies vary between 2 and more than 50 ng/mL [3,4,5,6,7,8] depending on the dose and the route of administration. However, to properly describe the elimination of this drug, the analytical method has to be reliable in concentrations below 1 ng/mL [9]. The ability to assess these low concentrations is essential for the development of a PK model that will allow for precise and well-informed predictions that may guide safe and efficient drug dosage in patients.

In the scientific literature, several analytical methods are described for BUP quantification in biological matrices. These approaches include radioimmunoassay [10], ELISA assay [11], cloned enzyme donor immunoassay (CEDIA) [12], gas chromatography coupled with mass spectrometry [13,14,15], as well as liquid chromatography coupled with UV [16] or fluorescent detectors [17]. Methods based on liquid chromatography and mass spectrometry [18,19] or tandem mass spectrometry [20,21,22,23,24] seem to provide the highest sensitivity. Nevertheless, almost uniformly the main issue reported by the authors was associated with achieving a satisfactory level of sensitivity. To overcome this limitation, different extraction methods were used, starting from the simple protein precipitation [23], liquid-liquid extraction [25,26,27], solid-phase extraction (SPE) [20,28,29], and “QuEChERS” techniques [30].

In the current study, we attempted to find a compromise between convenience, time efficiency, and cost optimization, on one hand, and high sensitivity due to the removal of matrix components on the other. Our main objective was to elaborate a high-throughput and sensitive analytical method and successfully utilize it in a densely sampled pharmacokinetic study in rabbits.

Since BUP is a highly lipid-soluble drug and a high degree of lipemia was expected in the study subjects, particular attention was paid to the accuracy of measurements under the condition of excessive lipid amounts in plasma.

Thus, the aim of our study was to develop a sensitive, repeatable, cost-efficient and easy HPLC analytical protocol for BUP in rabbit plasma, suitable for pharmacokinetic application under the conditions of a normal level of lipids as well as in lipemic samples. To confirm its suitability for PK studies, the method was tested during the preliminary pharmacokinetic trial on rabbits and the results are presented in this paper.

## 2. Results and Discussion

### 2.1. HPLC-MS^2^ Conditions

**Figure 1:**
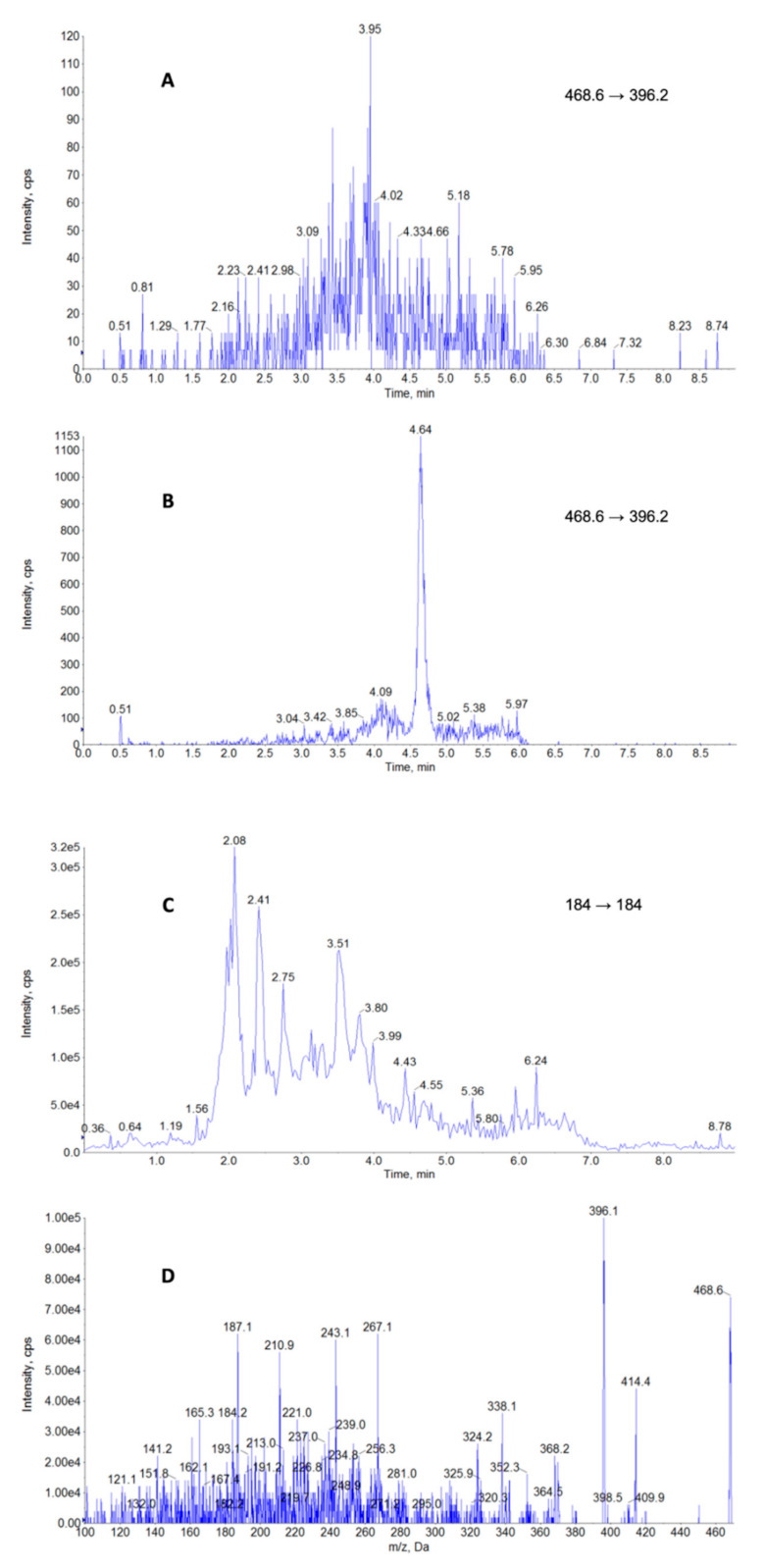
Example chromatograms of (**A**) blank plasma sample, (**B**) plasma sample containing buprenorphine residues at the limit of quantification (LOQ), (**C**) glycerophosphocholines (184 *m*/*z*), and (**D**) MS^2^ spectra of buprenorphine.

The chromatograms obtained after the method development and optimization of analytical conditions are shown in Figure 1. Panel A represents the chromatogram of a blank sample and indicates that no interference was observed, whereas panel B shows BUP at the limit of quantification (LOQ) level and proves a high sensitivity and a good peak shape.

BUP is a polar compound, therefore the electrospray ionization (ESI) mode was selected for the analysis [21,23,25,27,29,30,31]. All the parameters (declustering potential (DP), collision energy (CE), entrance potential) have been optimized with a direct infusion of working standard solutions (Table 1). Many authors pointed out the problem of the quality and stability of BUP ionization at low CEs (below 30 eV) [21,23,25,29,31]. To overcome that issue, one team [31] included trifluoroacetate ammonium in the mobile phase and obtained an improved ionization stability. Other authors monitored the precursor ion (468.6) as the highest intensity ion [21,23,25,29]. In our study, the optimization of BUP fragmentation at CEs above 30 eV allowed for satisfactory sensitivity and ionization stability (Figure 1D).

Most authors reported C18 columns as a stationary phase in BUP determination [23,25,27,29,30,31]. An alternative approach was proposed by Favretto et al. [21]. In order to improve the chromatographic separation and to shorten the retention time, the authors used a slightly polar phase column (cyanopropyl). This allowed for the use of a mixed reversed-phase and normal-phase separation mechanism [21]. In our work, the C18 column was used, but in order to accelerate the analysis and simultaneously obtain a good chromatographic separation a short 50 mm column with a small grain size of 2.6 µm was selected.

The mobile phases most frequently reported in the literature are ammonium formate or formic acid in combination with acetonitrile or methanol at acidic pH [21,23,25,26,29,30,31]. An alternative approach was proposed by Berg et al. [27], who used ammonium formate at pH 10.2. This method allowed for increased retention, improved peak shape, and increased analyte response. Additionally, the good removal of phospholipids was confirmed during the analysis. During the initial steps of method development, we used a high-pH liquid phase, as suggested by Berg et al. [27]. However, a loss of resolution, which indicated a very fast degradation of the column, was observed. Thus, we changed the phase to the 0.1% formic acid phase, which highly increased the column lifespan. 

Due to the high incidence of lipemic samples, the possibility of interference with phospholipids was closely monitored in our study. The monitoring of multiple reaction (MRM) indeed revealed the transition common for all representatives of these classes (184→184) (Figure 1C) [27,32,33]. To handle this issue, we developed a gradient which allowed for the clear chromatographic separation of phospholipids from BUP (Figure 1C) and contributed to the method’s low matrix effect (Table 2), as well as high repeatability and reproducibility (Table 3).

### 2.2. Extraction Procedure

The selected extraction procedure was an effect of tests aiming at the maximization of the drug recovery and the minimization of the matrix effect. In the literature, various solvents were used for liquid-liquid extraction, such as ethyl acetate [17]; methyl t-butyl ether:hexane [34]; n-butyl chloride:acetonitrile [15,22]; 1-chlorobutane:acetonitrile [35]; as well as commercially available Toxitubes A filled with a mixture of dichloromethane, 1,2-dichloroethane, heptane, and isopropanol [25]. The extraction described by Liu et al. [17] was especially important in our evaluations, as the authors used a very simple method to extract BUP from rabbit plasma obtained in a PK study. Despite a very similar application, this extraction could not be used directly in the case of samples from lipemic subjects. Such conditions need special handling, as the removal of lipids carries a high risk of insufficient extraction or variable extraction efficiency depending on the lipid content. Thus, during the development of our method, we investigated several extraction strategies and found out that the addition of hexane to the extraction solution provided a very stable recovery that was independent of the lipid concentration. Additionally, a comparably long time of extraction and duplication of the whole procedure was confirmed to significantly increase the drug signal.

In the current study, the apparent recovery for all concentration levels (0.25–2000 µg/L) was in the range of 98.7% to 109.0%. Compared to other works, this result is more than satisfactory. Authors who used simple protein precipitation [23,36] reported lower values (80–91%), which proves the very efficient drug extraction in the current protocol.

Interestingly, the calculated ion suppression of the matrix effect for BUP in plasma did not exceed 5.2% at the level of 2.5 µg/L, which is comparable to the more sophisticated and expensive “QuEChERS” purification technique [30]. Even though the proposed extraction protocol probably resulted in a substantial transfer of impurities, any interference that could result in loss of sensitivity was successfully omitted by the chromatographic separation (Figure 1).

### 2.3. Validation

The developed analytical procedure was validated according to the ICH Q2 (R1) method validation guidelines [37], which is a common procedure for validation in medical, toxicological, and pharmacokinetic analyses [32,38]. The validation parameters selectivity, linearity, repeatability, reproducibility, uncertainty, matrix effect, average recovery, screening detection limit (SDL), and limit of quantification (LOQ) were evaluated. The analysis of 20 blank samples of the matrices did not reveal any interference (as can be seen in Figure 1A), which confirmed the good selectivity of the method. The criteria concerning the relative retention time of the analytes corresponded to those of the calibration solution at a tolerance of ±2.5%. The linearity (R^2^) for all concentration levels (0.25–2000 µg/L) was 0.995, therefore the method may be considered linear in this range. For all the concentration levels (0.25–2000 µg/L), a repeatability of less than 6.4% (3.0–6.4%) and a within-laboratory reproducibility of below 9.3% (4.2–9.3%) were observed. The expanded uncertainty was calculated at the nine concentration levels, between 0.25 and 2000 µg/L, by applying a coverage factor of 2, which provided a level of confidence of approximately 95% (Table 3). The calculated average recovery and ion suppression of the matrix effect at the level 2.5 µg/L are reported in Table 2. The screening detection and quantification limits are presented in Table 2 as well.

### 2.4. Pharmacokinetic Study

**Figure 2:**
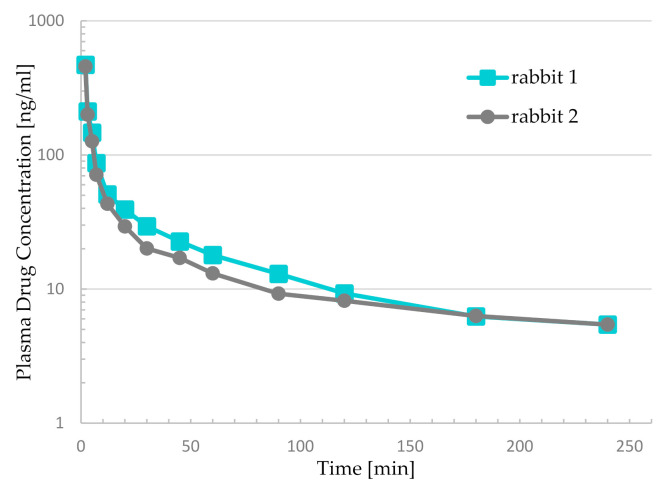
Buprenorphine concentration vs. time obtained for two rabbits after intravenous administration of 300 μg/kg.

To assess the suitability and applicability of the method, it was used to determine the concentrations of BUP in plasma samples collected from rabbits during a pilot pharmacokinetic study. The semilogarithmic plot of the concentration vs. time data from animals is presented in Figure 2 and the individual concentrations are provided in Table 4. Both rabbits had very similar PK profiles. All the samples showed concentrations above the LOQ, except those collected immediately before the drug administration. All the samples revealed that the drug levels were within the method linearity, and thus the selected range of the method was enough to comprehensively describe the PK profiles. The highest noted concentrations did not exceed 500 µg/L, and after an initial rapid decline the concentrations stabilized at levels below 10 µg/L. As the rabbits were subjected to general anesthesia, the study had to be limited to 4 h due to the risks associated with prolonged exposure to anesthetics. If the samples were taken for a longer time, the drug concentrations would certainly decrease. However, due to the high sensitivity of the analytical method, it is expected that buprenorphine would be detectable for several more hours.

In the present study, food was not withheld before the experiment. Due to the physiological specificity of the digestive tract in rabbits and the high risk of post-experimental complications, the animals could not be fasted before the PK experiment. This translated into significant postprandial lipemia and the risk of interference of the analytical method with lipids. However, a lack of lipid interference with IS or BUP was confirmed.

The comparison of our results with those in the literature indicates only minor differences. Three other authors [17,39,40] investigated the PK of BUP after intravenous administration to rabbits. Only one of them [39] used exactly the same dose of BUP (300 µg/kg) and similar experimental conditions as in our work. In this study, the highest concentrations were around 1000 µg/L (higher than in our study), and they declined rapidly with similar dynamics to those in the current work. The curve started to be flatter at around 90 to 120 min after BUP administration and, after that time-point, the concentrations remained below the level of 10 µg/L in both trials. The values noted at the time of the last sampling (240 min) were higher than 1 µg/L.

The slight differences observed between the existing literature and the present study may be caused by several factors, including the analytical method. However, due to numerous other discrepancies between the designs of these studies, such as in sampling times points, as well as in animals’ ages and weights and the concomitant use of anesthetic agents, the importance of the analytical differences seems to be difficult to assess. Additionally, the very small number of animals used in our pilot study limits any pharmacokinetic interpretations. Nevertheless, our goal was achieved, as the elaborated analytical method allowed us to fully describe the PK profiles, even during the elimination phase, and the results are in overall accordance with those of the published literature. Thus, the method was proven to be appropriate for the selected in vivo setting. However, a limitation of the presented method is that no active metabolites—namely, the bioactive norbuprenorphine and buprenorphine-3-glucuronide—were included in the analysis.

## 3. Materials and Methods

### 3.1. Reagents

Analytical grade buprenorphine was purchased from LGC Standards (Teddington, UK) and buprenorphine-D4 was purchased from Sigma-Aldrich (Darmstadt, Germany). Ethyl acetate (POCH, Gliwice, Poland), n-hexane (Merk, Darmstadt, Germany) and ammonia solution 25% (Stanlab, Lublin, Poland) were of HPLC or analytical grade. Formic acid and methanol were from Sigma-Aldrich (Darmstadt, Germany) and were of analytical grade and LC-MS grade, respectively.

The plasma samples used in the method development and validation were obtained from rabbits involved in the pharmacokinetic/pharmacodynamic study. All blank plasma samples were obtained from animals which did not receive any drugs except isoflurane used in inhalatory anesthesia.

### 3.2. Standard Solutions

BUP and BUP-D4 primary standard stock solutions were dissolved in water/methanol (1:1, *v*/*v*) and stored at −70 °C for less than 1 year. The stock solutions were further dissolved to working solutions using the same water/methanol mixture.

### 3.3. High-Performance Liquid Chromatography–Tandem Mass Spectrometry

The HPLC-MS^2^ system consisted of an ABSciex Exion LC HPLC system connected to ABSciex API 5500 Qtrap mass spectrometer (AB Sciex, Concord, ON, Canada). The Analyst 1.6.3 software controlled the HPLC-MS^2^ system and Multiquant 3.2 was used to process the data. The mass spectrometer was operated in the positive ESI mode with a capillary voltage of 5.5 kV. The temperature of desolvation was set at 500 °C, gas 1 (air)—45 psi; gas 2 (air)—45 psi; collision gas (N2)—medium; nebuliser gas (N2)—40 psi; curtain gas (N2)—35 psi. The voltage of the electron multiplier was set at 2.2 kV. The chromatography was performed in a Kinetex^®^ 2.6 µm XB-C18 column 50 × 2.1 mm (Phenomenex, Torrance, CA, USA), connected to a SecurityGuard™ ULTRA C18 2.1 mm precolumn (Phenomenex, Torrance, CA, USA). The mobile phase was composed of two solutions: A (0.1% formic acid phase) and B (methanol) in a gradient mode which started with 5% of B. From 0.5 to 1 min, the concentration of B was raised to 30%, then from 1 to 3 min, the concentration of B was raised to 90% and left for 2 min. Finally, after that the B concentration was decreased from 5 to 6 min to 5% and left for 1 min. The flow rate was 0.4 mL/min. The column was thermostated at 40 °C. The ions were monitored in MS^2^ mode (Table 1). The mass spectrometry parameters for the internal standard were as follows: the ions monitored by MRM were 472.15→400.2. The DP was 196 eV. The optimized CE for the internal standard was 53 eV for the first ion transition.

### 3.4. Validation

The analytical method validation was carried out according to the previously mentioned ICH Q2 (R1) method validation guidelines [37]. Selectivity, linearity, uncertainty, precision, SDL, and LOQ were evaluated during this procedure. Analyte standard solutions at different concentrations—0.25, 0.5, 2.5, 10, 40, 100, 500, 1000, 2000 µg/L—were added to the blank sample containing an internal standard (25 µg/L), then subjected to the liquid-liquid extraction and HPLC-MS^2^ procedure. The analyte peak area was plotted against the corresponding concentrations and the calibration curves were set up by means of the least-squares method. SDL and LOQ were estimated by calculations based on the signal-to-noise ratio. The determination of the signal-to-noise ratio was performed by comparing the measured signals from samples with known low concentrations of analyte with those of blank samples and establishing the minimum concentration at which the analyte can be reliably detected or quantified. A typical signal-to-noise ratio is 3:1 for SDL and 10:1 for LOQ. Spiked blank samples were prepared as follows: standard solutions of different concentrations corresponding to 0.25, 0.5, 2.5, 10, 40, 100, 500, 1000, and 2000 µg/L and internal standards (buprenorphine—D4) corresponding to 25 µg/L were added to 200 µL of mixed plasma sample. Spiked blank samples were analyzed according to the previously described procedure. The repeatability and reproducibility were determined at nine concentration levels (six samples of each level) 0.25, 0.5, 2.5, 10, 40, 100, 500, 1000, and 2000 µg/L. For repeatability, the samples were analyzed by the same operators on the same day with the same instrument, and were calculated as the relative standard deviation (RSD, %). For within-laboratory reproducibility, another two sets of blank samples were fortified and analyzed by different operators on another two days with the same instrument, and were calculated as the relative standard deviation (RSD, %).

In selectivity study, possible interferences encountered in the method have been checked by analysis of 20 blank samples for each matrix from different sources. The recovery was calculated by comparing the mean measured concentration with the fortified concentration of the samples. The matrix effect was checked by analyzing five different samples at 2.5 µg/L concentration level, which is ten times higher than the LOD level for this method and calculated by the equation proposed previously by Matuszewski [41].

The expanded uncertainty was calculated at the nine concentration levels, corresponding to 0.25, 0.5, 2.5, 10, 40, 100, 500, 1000, and 2000 µg/L by applying a coverage factor of 2, which gave a level of confidence of approximately 95% [42].

### 3.5. Sample Preparation

Two hundred microliters of blank rabbit plasma or plasma fortified with BUP was spiked with 100 μL of the IS solution (to obtain the concentration of 25 μg/L), and 50 μL of 25% ammonia solution (*v*/*v*). After a brief manual shaking, 1200 μL of hexane/ethyl acetate (1:1 *v*/*v*) was added. The samples were shaken on a horizontal shaker for 30 min, centrifuged for 8 min in 20 000 g, and then frozen. After the solidification of the aqueous part of the sample, the organic layer was transferred to clean tubes, and the extraction procedure was repeated. The combined organic fraction was evaporated until dryness in a vacuum concentrator (Eppendorf, Hamburg, Germany; vacuum mode, 30 °C). The residues were redissolved in 150 μL of methanol/water (1:1 *v*/*v*), filtered through Nanosep MF 0.2 μm (Pall Laboratory, New York City, USA), and transferred into vials.

### 3.6. Pharmacokinetic Evaluation

The study was a pilot part of the experiment that was approved by the Local Animal Experimentation Committee in Wrocław (permit number 42/2017). All the procedures complied with the local and international law and regulations, and all efforts were made to reduce animal distress and suffering. During the experiment, two male New Zealand rabbits weighing 3.679 and 3.882 kg were kept in general anesthesia, induced and maintained by a constant flow of isoflurane. The animals were intubated with an endotracheal tube and catheters were placed to the marginal ear vein and the marginal ear artery. After that, the animals were handled on a stable minimal alveolar concentration (ranging between 1.5 and 1.7 MAC (minimal alveolar concentration) of isoflurane for 240 min. BUP was administered to the marginal ear vein at a dose of 300 µg/kg body weight. Blood (600 µL) was collected to heparinized tubes from the marginal ear artery before the drug administration and at 2, 3, 5, 7, 12, 20, 30, 45, 60, 90, 120, 180, 240 min after the drug administration. During the first 35 min of dense blood sampling, the animals were administered with normal saline (constant infusion, a total volume of 8.75 mL/kg). The collected blood samples were centrifuged (5000 rpm, 5 min) and separated plasma was transferred to fresh tubes and stored at −70 °C until the time of analysis.

## 4. Conclusions

The developed and validated HPLC-MS^2^ method meets the criteria set for analytical procedures that can be used in pharmacokinetic analyses. The validation outcome indicated a high repeatability and reproducibility, with an average recovery of between 98.7% and 109%. The method was proven to be linear, highly selective, and sensitive. Additionally, the reported pharmacokinetic profiles in rabbits provide evidence that the presented analytical method can be successfully applied to the in vivo PK studies, where clinically relevant concentrations of BUP and its metabolites often fall into the low nanogram range.

## Figures and Tables

**Table 1:** Precursor ions and fragment ions of buprenorphine.

Analyte	Precursor Ion (m/z)	Ion Transition (m/z)	Declustering Potential (eV)	Entrance Potential (eV)	Collision Energy (eV)
Buprenorphine	468.6	396.2 414.2	196 196	10 10	53 45

**Table 2:** Validation report for buprenorphine in rabbit plasma.

Parameter	Value
SDL [µg/L]	0.08
LOQ [µg/L]	0.25
Matrix Effect [%]	5.2 ± 2.1
Concentration Range [µg/L]	0.25–2000
Determination Coefficient	0.995
Calibration Curve	y = 19553x + 12072
Recovery [%]	98.7–109.0

SDL—screening detection limit; LOQ—limit of quantification.

**Table 3:** Parameters obtained for the calibration curve.

Level	Repeatability (RSD, %) (*n* = 6)	Within-Lab Reproducibility (RSD, %) (*n* = 18)	Apparent Recovery (%)	Expanded Uncertainty (µg/L)
0.25 µg/L	6.4 ± 4.1	9.3 ± 5.1	109.0 ± 4.8	0.25 ± 0.06
0.5 µg/L	5.8 ± 3.6	7.6 ± 4.4	106.3 ± 2.9	0.5 ± 0.10
2.5 µg/L	4.9 ± 3.1	7.1 ± 4.5	101.9 ± 3.8	2.5 ± 0.44
10 µg/L	4.6 ± 3.5	6.2 ± 4.1	104.0 ± 2.8	10 ± 1.60
40 µg/L	4.1 ± 3.1	6.4 ± 3.8	102.6 ± 3.8	40 ± 6.40
100 µg/L	3.6 ± 3.4	5.1 ± 3.6	103.5 ± 3.2	100 ± 12.8
500 µg/L	3.3 ± 2.9	4.9 ± 3.4	101.0 ± 4.2	500 ± 61.3
1000 µg/L	3.0 ± 2.8	4.7 ± 3.3	99.0 ± 4.2	1000 ± 117.5
2000 µg/L	3.1 ± 2.5	4.2 ± 3.0	98.7 ± 6.3	2000 ± 200.0

RSD—relative standard deviation.

**Table 4:** Concentrations of buprenorphine evaluated for two rabbits during the pharmacokinetic experiment.

Time (min)	Concentration Rabbit 1 (µg/L)	Concentration Rabbit 2 (µg/L)
2	456.68	470.01
3	199.81	211.07
5	126.41	146.52
7	71.21	86.95
12	43.24	50.77
20	29.28	39.14
30	20.07	29.35
45	17.05	22.61
60	13.07	17.92
90	9.23	12.97
120	8.18	9.27
180	6.30	6.25
240	5.43	5.44

## Data Availability

All data is contained within this article.

## Sample Availability

Samples of the compounds are not available from the authors.
